# Foodborne Disease Outbreaks Linked to Foods Eligible for Irradiation, United States, 2009–2020

**DOI:** 10.3201/eid3006.230922

**Published:** 2024-06

**Authors:** Marta Zlotnick, Taylor Eisenstein, Misha Park Robyn, Katherine E. Marshall

**Affiliations:** Oak Ridge Institute for Science and Education, Oak Ridge, Tennessee, USA (M. Zlotnick);; Centers for Disease Control and Prevention, Atlanta, Georgia, USA (M. Zlotnick, T. Eisenstein, M.P. Robyn, K.E. Marshall)

**Keywords:** Food irradiation, *Campylobacter*, *Listeria monocytogenes*, *Salmonella*, *Escherichia coli*, food safety, foodborne diseases, disease outbreaks, bacteria, United States

## Abstract

Food irradiation can reduce foodborne illnesses but is rarely used in the United States. We determined whether outbreaks related to *Campylobacter*, *Salmonella*, *Escherichia coli*, and *Listeria* *monocytogenes* were linked to irradiation-eligible foods. Of 482 outbreaks, 155 (32.2%) were linked to an irradiation-eligible food, none of which were known to be irradiated.

Food irradiation has been studied globally for decades and is a safe, effective means of reducing foodborne illness–causing pathogens, sterilizing insects, delaying ripening or sprouting, and extending shelf life (*1*,*2*). The US Food and Drug Administration has approved various foods for irradiation, including meat, poultry, fresh shell eggs, and spices (*2*) (Appendix Table). However, irradiation has not been widely adopted in the United States because of large fixed costs and the perception of consumer unwillingness to purchase irradiated food (*3*). Estimates of the amount of irradiated food available in the United States are scarce, but as of 2010, approximately one third of spices consumed and <0.1% of imported fruit, vegetables, and meats were irradiated (*3*).

*Campylobacter*, *Salmonella*, *Escherichia coli*, and *Listeria monocytogenes* are among the most common bacterial foodborne pathogens causing illnesses, hospitalizations, and death in the United States (*4*) and can be neutralized by irradiation at sufficient doses (*5*). We identified outbreaks caused by these pathogens and linked to irradiation-eligible foods; then, we determined whether any of the foods had been irradiated.

In the United States, the Foodborne Disease Outbreak Surveillance System (FDOSS) collects information from state, local, and territorial health departments about foodborne disease outbreaks. The National Outbreak Reporting System, launched in 2009, reports information gathered by FDOSS, including food processing methods such as shredding, pasteurizing, or irradiation. We searched for foodborne disease outbreaks reported and finalized through FDOSS and the National Outbreak Reporting System as of February 4, 2022, for which the date of first illness onset occurred during 2009–2020 and a confirmed pathogen was *Campylobacter*, *Salmonella*, *E. coli*, or *Listeria monocytogenes.* A foodborne disease outbreak was defined as >2 illnesses linked to a common exposure with evidence suggesting a food source. FDOSS variables we examined included method of processing, food vehicle, Interagency Food Safety Analytics Collaboration (IFSAC) food category, and the number of estimated primary illnesses, hospitalizations, and deaths. We grouped outbreaks by IFSAC category and irradiation approval status (eligible, some foods eligible, not yet eligible, or undetermined) (Appendix Table). We conducted a literature review to identify outbreaks not captured through FDOSS. We obtained foods approved for irradiation for pathogen reduction and approval years from the Code of Federal Regulations 21 Part 179 (Appendix Table).

In FDOSS, we identified 2,153 foodborne outbreaks during 2009–2020 caused by *Campylobacter*, *Salmonella*, *E. coli*, or *Listeria monocytogenes*. Of those, 482 (22.4%) included information regarding processing methods other than unknown or a missing value; none had irradiation listed as a processing method. Of the 482 outbreaks, 155 (32.2%) were linked to a food eligible for irradiation when the onset of the first reported illness occurred; those outbreaks resulted in 3,512 illnesses, 463 hospitalizations, and 10 deaths (Appendix Table). The most common sources were chicken (52 outbreaks), beef (31), and eggs (29), comprising 72% (112/155) of outbreaks linked to irradiation-eligible foods.

During our literature search, we identified 1 outbreak linked to food that might have included an irradiated ingredient. During 2009–2010, *Salmonella enterica* serotype Montevideo was found in imported pepper used in ready-to-eat salami (*6*). Some of the manufacturer’s pepper was reportedly irradiated, but some was not. Whether the implicated product contained irradiated pepper is unclear. Irradiation was not reported as a processing method for the outbreak in FDOSS. After consultation with the Centers for Disease Control and Prevention outbreak investigation team, we determined there was insufficient evidence to link that outbreak to irradiated pepper.

The illnesses, hospitalizations, and deaths associated with outbreaks linked to irradiation-eligible foods might have been prevented or reduced had these foods been irradiated. Irradiation has repeatedly been proposed as a strategy to reduce foodborne disease outbreaks (*5*,*7*,*8*). Irradiation typically eliminates a large proportion of pathogenic microorganisms. The efficacy of irradiation depends on factors like temperature and water content (*9*). Food may become contaminated after irradiation. Irradiation can be a useful tool in improving food safety complementary to existing food safety practices. Consumer demand for irradiated foods may be increased through education (*10*).

The first limitation of our study is that IFSAC food categories do not always correspond to food groups approved for irradiation by the US Food and Drug Administration (Appendix Table); therefore, misclassification of irradiation approval status might have occurred for some foods. Reporting of outbreaks to FDOSS is voluntary, and processing method information was frequently missing, so irradiation might have been underreported or unrecognized by public health partners because of limited knowledge of irradiation or unfamiliarity with labeling. For outbreaks with multiple etiologies including a pathogen other than the 4 of interest, irradiation might not have reduced those pathogens.

We identified 155 *Campylobacter*, *Salmonella*, *E. coli*, or *Listeria* *monocytogenes* outbreaks with a known method of processing that were linked to irradiation-eligible foods during 2009–2020; none of the implicated foods were reported as irradiated. These results suggest that some outbreaks could be prevented or mitigated through irradiation. Prioritizing food irradiation efforts, particularly for chicken, beef, and eggs, could substantially reduce outbreaks and illnesses.

Appendix Additional information about foodborne disease outbreaks linked to foods eligible for irradiation, United States, 2009–2020
