# Supplementary material for: Foodborne Disease Outbreaks Linked to Foods Eligible for Irradiation, United States, 2009–2020
Source: Emerg Infect Dis. 2024 Jun;30(6):1291–3. doi: 10.3201/eid3006.230922 (PMC11138973; doi:10.3201/eid3006.230922)
Supplement: Appendix — Additional information about foodborne disease outbreaks linked to foods eligible for irradiation, United States, 2009–2020 [file 23-0922-Techapp-s1.pdf]

Article DOI: https://doi.org/10.3201/eid3006.230922

EID cannot ensure accessibility for supplementary materials supplied by authors. Readers who have difficulty accessing supplementary content should contact the authors for assistance.

Foodborne Disease Outbreaks Linked to Foods Eligible for Irradiation, United States, 2009–2020

Appendix

Appendix Table. Foodborne outbreaks of confirmed *Campylobacter, Salmonella, E. coli,* and *Listeria* infections* with a method of processing reported to the Foodborne Disease Outbreak Surveillance System (FDOSS), 2009–2020, by food irradiation eligibility status, and Interagency Food Safety Analytics Collaboration (IFSAC) food category (*1*) (n = 482)

IFSAC commodity category of implicated food	No. of outbreaks	Estimated primary illnesses	Number of hospitalizations	Number of deaths	Year FDA approved for irradiation for microorganism control in food (*2*)
Eligible for irradiation					
Beef	31	427	118	2	1997 (meat) †
Chicken	52	1055	112	1	1990 (poultry) ‡
Crustaceans§	5	80	22	0	2014
Eggs	29	556	64	3	2000
Mollusks	4	25	3	0	2005
Other Meat¶	1	3	1	0	1997 (meat) †
Other Poultry#	2	5	1	0	1990 (poultry) ‡
Pork	19	674	119	4	1997 (meat) †
Turkey	12	687	23	0	1990 (poultry) ‡
Total, food categories eligible for irradiation	155	3512	463	10	
Some foods eligible for irradiation**					
Herbs	5	158	28	0	1986 (dried/dehydrated spices and herbs)
Vegetable Row Crops	22	777	268	5	2008 (fresh iceberg lettuce and fresh spinach)
Total, food categories with some foods eligible for irradiation	27	935	296	5	
Not yet eligible for irradiation					
Dairy	133	1625	303	19	Not currently approved
Fish	4	138	7	0	Not currently approved
Fruits††	30	786	271	11	Not currently approved
Fungi	1	55	6	0	Not currently approved
Game	1	33	18	0	Not currently approved
Grains-Beans	3	246	46	0	Not currently approved
Nuts-Seeds	3	89	9	0	Not currently approved
Oils-Sugars	1	7	1	0	Not currently approved
Roots/ Underground	3	1398	215	0	Not currently approved

IFSAC commodity category of implicated food	No. of outbreaks	Estimated primary illnesses	Number of hospitalizations	Number of deaths	Year FDA approved for irradiation for microorganism control in food (2)
Seeded Vegetables	9	311	98	0	Not currently approved
Sprouts	7	352	33	1	Not currently approved
Total, food categories not yet eligible for irradiation	195	5040	1007	31	
Undetermined eligibility for irradiation‡‡					
Multiple	88	2050	310	4	n/a
Other	3	251	57	0	n/a
Unknown, unreported, or unclassifiable	14	228	39	5	n/a
Total, food categories with undetermined eligibility	105	2529	463	9	
Total, All	482	12016	2172	55	

* Six out of 482 outbreaks included a confirmed pathogen of interest and another confirmed pathogen other than the 4 of interest. The other pathogens in these 6 outbreaks were *Bacillus*, *Clostridium*, *Cryptosporidium*, Norovirus, and *Staphylococcus*. One of the 6 outbreaks involved an irradiation-eligible food;
† 21 CFR Part 179 (December 3, 1997) indicates that, for the purposes of the rule, meat sources include cattle, sheep, swine, or goats;
‡ 21 CFR Part 179 (May 2, 1990) states that, for the purposes of the rule, poultry sources include "any domesticated bird, including chickens, turkeys, ducks, geese, or guineas.";
§ The five outbreaks linked to crustaceans took place in 2014 or later;
¶ In the IFSAC scheme, Other Meat refers to meat that is not beef or pork, such as goat;
# In the IFSAC scheme, Other Poultry refers to poultry that is not chicken or turkey, such as duck;
** Only specific foods within the herbs and vegetable row crops categories are approved for food irradiation. Food vehicle information needed to determine eligibility was not available for all outbreaks; therefore, these were classified as "some foods eligible." Outbreaks linked to foods eligible for irradiation may be underestimated;
†† Fruit is eligible for irradiation for the purposes of disinfestation of insects; this is overseen by the U.S. Department of Agriculture Animal and Plant Health Inspection Service (USDA-APHIS). Irradiation Phytosanitary Treatment of Imported Fruits and Vegetables, 7 CFR Parts 305 and 319 (Oct. 23, 2002);
‡‡ Records for these outbreaks did not include enough information to determine whether they were caused by foods eligible for irradiation.

References

1. Richardson LC, Bazaco MC, Parker CC, Dewey-Mattia D, Golden N, Jones K, et al. An updated scheme for categorizing foods implicated in foodborne disease outbreaks: a tri-agency collaboration. Foodborne Pathog Dis. 2017;14:701–10. PubMed https://doi.org/10.1089/fpd.2017.2324

2. US Food and Drug Administration, Health and Human Services. Irradiation in the production, processing and handling of food, 21 CFR part 179. 2024. [cited 2023 Jan 18]. https://www.ecfr.gov/current/title-21/chapter-I/subchapter-B/part-179
